# Closing the sensory feedback loop is necessary for effective neurorehabilitation

**DOI:** 10.1371/journal.pbio.3002866

**Published:** 2024-10-29

**Authors:** Andrea Cimolato, Stanisa Raspopovic

**Affiliations:** Center for Medical Physics and Biomedical Engineering, Medical University of Vienna, Vienna, Austria

## Abstract

Recent advances in neurotechnology enable somatosensory feedback restoration in disabled individuals. This Perspective discusses how closing the sensory feedback loop in brain implants and nerve electrodes for stimulation may improve rehabilitation and assistive systems for patients.

## Teaser

Recent advances in neurotechnology enable somatosensory feedback restoration in disabled individuals. This Perspective discusses how closing the sensory feedback loop with brain implants and nerve electrodes for stimulation may improve rehabilitation and assistive systems for patients.

Somatosensations are essential for interacting with the environment and coordinating our movements to perform daily activities. These sensory inputs deliver protective information to the brain about the dangers when touching hot surfaces, or positions and forces exerted by our body, for example [1]. Touch and proprioceptive feedback are vital for complex tasks like walking on uneven surfaces in darkness or precise hand–object interactions while performing surgical operations, to ensure smooth and effective motor control. In addition, affective touch, during holding or cuddling, is crucial for emotional and social well-being, providing key sensory inputs that regulate physiological responses, such as stress hormone levels and supporting healthy brain development, enabling improvements in stress and overall mood [2].

**Fig 1:**
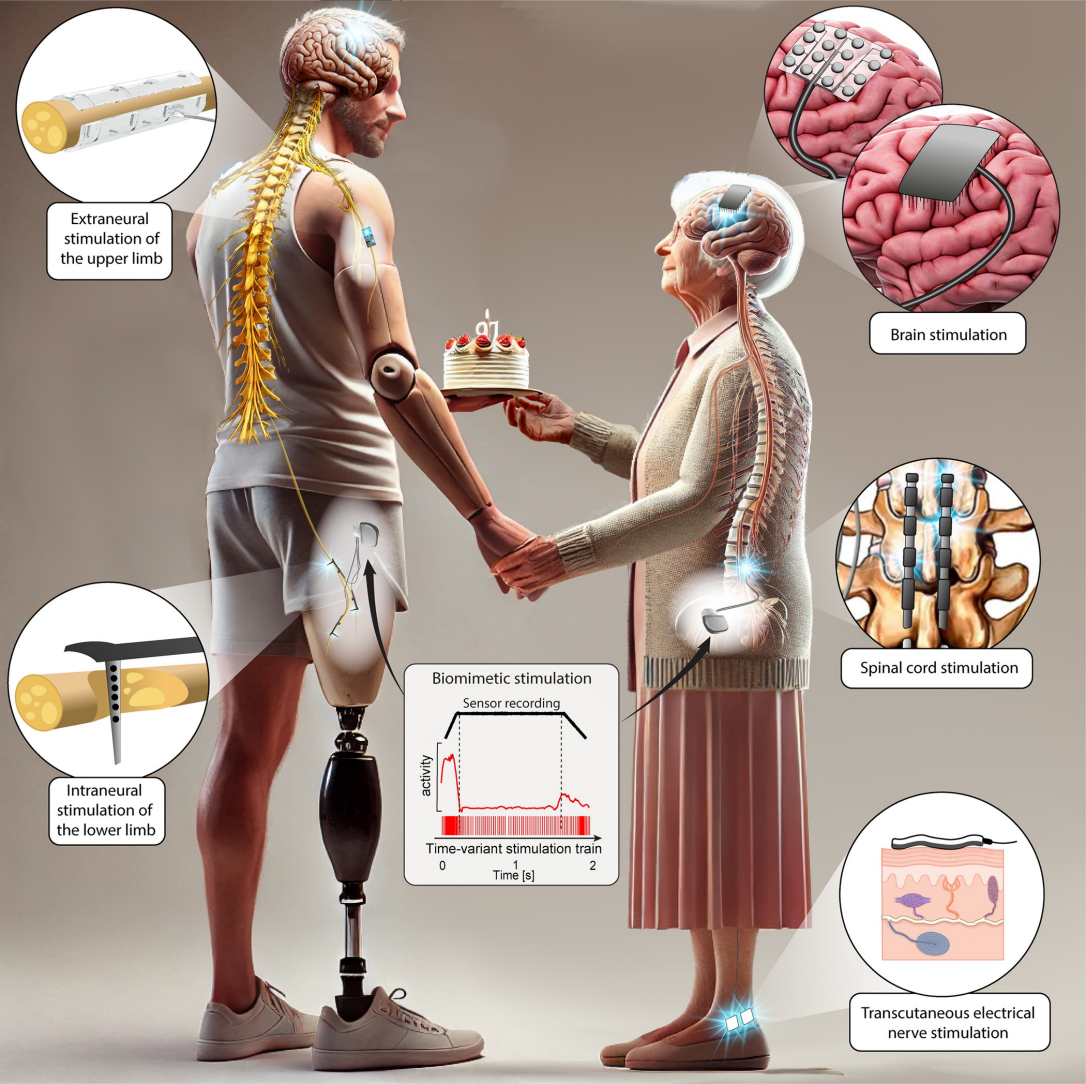
Somatosensory neural interfaces used to restore everyday actions. On the left, an individual with upper and lower limb amputations, is shown with extraneural and intraneural peripheral nerve interfaces, restoring touch. On the right, an elderly person suffering from neurological disorders is shown. Somatosensory stimulation of the brain, spinal cord, and peripheral nerves are illustrated, restoring sensory feedback from the hands and legs. The biomimetic algorithms transforming artificial sensors’ signals into the policy of electrical stimulation are shown in the middle, which are implemented within the implantable stimulators. (Some elements of the figure were created using ChatGPT artificial intelligence software for image generation).

Both able-bodied and disabled individuals during rehabilitation tend to underestimate the importance of sensations during movement, because feedback is naturally integrated in each task. Sensory feedback is interrupted or degraded in many neurological disorders, such as amputations, neuropathies, stroke, or spinal cord injury (SCI). Neural interfaces, connecting directly with the residual somatosensory pathway, offer a promising solution by leveraging the nervous system’s natural signals to recreate quasi-natural sensations. This allows users to effectively incorporate sensory inputs into their body schema, potentially accelerating the rehabilitation process and improving recovery. Somatosensory neuroprostheses can be used to target different locations within the neural pathway, including the skin, peripheral nerves, spinal cord, and somatosensory cortex (Fig 1).

Direct brain stimulation interfaces with the brain at the cortical level, particularly within the primary somatosensory cortex. This technique typically involves the implantation of intracortical microelectrode arrays beneath the dura mater. These interfaces have shown significant potential in clinical settings, where they have been used to recreate artificial tactile sensations in disabled individuals [3] and to substantially improve functionality of human quadriplegic individuals during motor tasks [4]. Restoring sensations directly to the brain has the potential to produce natural-feeling experiences, while not suffering from the excessive displacements due to interface movement. However, it comes at the cost of the invasive nature of these implants and their instability over time due to scarring. In addition, considering inherent limits to the number of physical variables (position, intensity, etc.) that can be encoded via electrostimulation, the correct choice of those which are most functional is not trivial. Indeed, solving interface stability, while minimizing the surgery complexity, is a high-risk high-gain goal of many research and industrial efforts worldwide, raising hope that these neuroprotheses could soon become the clinical reality.

Downstream, the possible treatment for less severe SCIs, stroke, or even amputations is spinal cord stimulation. This involves the insertion of commercially available multi-channel leads into the epidural space on the dorsal side of the spinal cord to provide artificial sensory restoration. This has recently been used to successfully recreate artificial sensations for lower limb amputees [5], enhancing their rehabilitation by decreasing their pain while increasing their movement ability. However, despite being clinically approved and showing some success in evoking useful sensations and functional benefits, it often results in paraesthesia (e.g., tingling or prickling) or less localized sensations; such unpleasant sensations could potentially be the trigger for device abandonment, and this is an open problem yet to be solved.

Traveling further downstream in the nervous system, peripheral nerve interfaces (PNIs) are the next natural target; these interfaces are designed to restore sensations by either wrapping around the nerve or being inserted via intraneural injection [6]. PNIs are often used to treat both upper and lower limb amputees, providing sensory feedback that significantly enhances prosthesis motor control, allowing users to regulate grasping force with precision and even identify object characteristics through real-time tactile input [6]. The use of PNIs results in overall prosthesis use increase [7], reducing the likelihood of prosthetic abandonment. In lower limb amputees, PNIs recreate tactile sensations from the amputated leg, enabling users to reintegrate information about stance force and swing motion during walking, leading to increased mobility [6]. Health benefits for upper and lower limb amputees include a reduction in phantom limb pain and sensory distortions, which could be pivotal for the reimbursement of devices from health insurance in the future. A primary challenge with PNIs, however, is the inconsistency of the restored sensations over time, largely due to the instability of the interfaces, hindering their long-term usability.

To minimize the risks associated with surgical interventions, transcutaneous electrical nerve stimulation (TENS) is a noninvasive alternative designed to stimulate peripheral nerves through the skin. Although the quality of sensations provided by TENS is less somatotopic and natural compared to invasive methods, they can provide some functional benefits [8]. This approach offers a promising alternative, especially for peripheral neuropathies, by maintaining some clinical improvements while reducing risks related to surgical procedures. However, current TENS devices face significant challenges in daily usability, including frequent recalibration due to unstable skin-electrode interfaces and due to movement or sweating. Automatized recalibration with artificial intelligence (AI)-based approaches hold promise to address these issues.

The question remains, how do these technologies fill the sensory gap, and which ones are most effective for each specific patient category? The choice of an optimal interface technology depends on the specifics of an individual’s disability and their personal perception regarding the trade-off between the invasiveness and naturalness of the sensations elicited. For instance, a quadriplegic individual (i.e., high level SCI) would have to opt for brain implants, since the distal neural structures are nonresponsive, while diabetics with peripheral loss could opt for the TENS since their proximal nerve is functional and their skin can have problematic healing, thus favoring a noninvasive approach. Another consideration is that neural interfaces still fall short in reproducing the intricate patterns of neural activation needed to create natural-like sensations. To address these challenges, biomimetic approaches have emerged as a key strategy, focusing on mimicking the brain’s natural processes to achieve more natural feedback [9]. Together, the choice of the optimal combination of neurotechnology and surgical approach should be done by multidisciplinary teams considering the risk/benefits ratio of described techniques.

Although described technologies have proven potential for assistance or rehabilitation, they are yet to be adopted in clinical settings. To that aim, we note the relevance of creating a more holistic protocol that addresses not only motor but especially (multi)sensory inputs, and cognitive aspects of how the impaired body part is perceived and self-represented [10]. Emerging technologies, like virtual reality can create customizable and controlled 3D environments, enhancing personalized rehabilitation, by targeting patient-particular sensory and motor functions. Their combination with neural interfaces for sensory feedback restoration holds potential to create a more immersive and controlled rehabilitation experience for patients.

There is no doubt that the integration of AI and miniaturized wearable technologies will enhance the effectiveness of feedback, enabling real-time optimization of stimulation parameters and customizable data-driven therapies. Realistically, the broader use of these innovations in healthcare should occur within the next 10 years. Bionic solutions utilizing these innovations will enable a new era that we anticipate will significantly improve patients’ quality of life and open the door to their complete recovery.
